# Improved Corrosion Resistance of Magnesium Alloy in Simulated Concrete Pore Solution by Hydrothermal Treatment

**DOI:** 10.1155/2020/4860256

**Published:** 2020-09-07

**Authors:** Ye Wang, Guosong Wu, Jiapeng Sun

**Affiliations:** College of Mechanics and Materials, Hohai University, Nanjing 211100, China

## Abstract

Magnesium alloys are considered for building materials in this study due to their natural immunity to corrosion in alkaline concrete pore solution. But, chloride ions attack often hinders the application of most metals. Therefore, it is necessary to conduct a preliminary corrosion evaluation and attempt to find an effective way to resist the attack of chloride ions in concrete pore solution. In our study, hydrothermal treatment is carried out to modify Mg-9.3 wt. % Al alloy. After the treatment in NaOH solution for 10 h, scanning electron microscopy (SEM) reveals that a layer of dense coating with a thickness of about 5 *μ*m is formed on Mg alloy. Energy dispersive X-ray spectroscopy (EDS), X-ray photoelectron spectroscopy (XPS), and X-ray Diffraction (XRD) are combined to analyze the coating, and it is thereby confirmed that the coating is mainly composed of Mg(OH)_2_. As expected, both immersion test and electrochemical corrosion test show that the coated magnesium alloy has a better corrosion resistance than the uncoated one in simulated concrete pore solution with and without chloride ions. In summary, it indicates that hydrothermal treatment is a feasible method to improve the corrosion resistance of Mg alloys used for building engineering from the perspective of corrosion science.

## 1. Introduction

Rapid corrosion in aqueous solutions always hampers the applications of magnesium alloys in the automotive, aerospace, electronics industry, and biomedical field [1–6]. Nowadays, the concept of lightweight construction and equipment has been proposed for overcoming the energy and resources shortage in the development of our society [7–9]. Therefore, magnesium alloys as one of the lightest structural materials are still very promising in the future industries. Concrete is one of the most important building materials in civil engineering, and usually, it is always strengthened by steel bars for improving its poor tensile strength [10]. Although steel has many advantages over other metals, it is always anticipated based on the consideration of energy-saving that there will be a lighter reinforcement bar for fully or partly replacing steel bars. Bamboo has a much lower density than steel, which has already been attempted in civil engineering in recent years. But, insect and fungus attacks, shrinking, and swelling are big disadvantages [11, 12]. Aluminum alloys are also regarded as an alternative to steels in reinforced concretes due to their low density and noncorrosive characteristics, but their native oxide coating will become unstable at acid or alkaline environments, inducing aluminum alloy bars susceptible to corrosion in the alkaline environment of concrete constructions [13]. The density of magnesium is only two-thirds that of aluminum and one-fourth that of iron and the specific strength of magnesium is higher than that of iron or aluminum [14]. Obviously, it may attempt to select magnesium alloys for reinforcement bars in some low-load bearing constructions according to the strengthening rule of reinforced concrete. Furthermore, Mg-H_2_O diagram tells that magnesium has a good immune behavior in alkaline aqueous environments [15, 16]. Thus, it may predict that magnesium and its alloys can survive in those alkaline concrete pore solutions. In addition, magnesium is one of the most abundant elements in the earth's crust and oceans [17, 18]. Therefore, it seems significant to conduct a preliminary investigation on the corrosion behavior of magnesium alloys in construction environments.

It is well known that the chloride-induced corrosion of reinforcing steel is one of the most common damage phenomena of steel-reinforced concrete structures, particularly in the coastal marine environment [19, 20]. Therefore, it must be carefully considered when magnesium alloys are proposed for these new applications in building engineering. Now, alloying and surface treatment are two main ways to improve the corrosion resistance of magnesium alloys, and the latter is more economical [14, 21]. Usually, a surface barrier layer can isolate the bulk materials from the external environment to avoid corrosion. However, magnesium is a very active metal in the galvanic series inducing galvanic corrosion when it is in electrical contact with many other conductive materials in the same electrolyte [22, 23]. According to the theory of galvanic corrosion, if defects such as pores and cracks occur in those electroconductive coatings, galvanic corrosion will happen when the electrolyte reaches the interface between the coating and substrate *via* these defects [22, 23]. In general, insulating materials are favorite in those coatings on Mg alloys from the perspective of corrosion protection.

In recent years, hydrothermal treatment has been attempted to prepare Mg(OH)_2_ or layered double hydroxide (LDH) coatings on Mg alloys for improving the corrosion resistance in sodium chloride solutions [24–27]. In our study, this method was selected for the surface treatment of Mg-Al alloys, and the corrosion behavior after the treatment was investigated in simulated concrete pore solutions with chloride ions.

## 2. Materials and Methods

As-cast Mg-9.3 wt. % alloys were used for substrate materials in this investigation, and the samples were cut into 10 mm × 10 mm × 5 mm pieces. The samples were mechanically ground by up to #1200 emery paper and then polished with Al_2_O_3_ paste. Next, they were ultrasonically washed in ethanol for 5 min and dried prior to hydrothermal treatment. Each sample was laid at the bottom of 25 ml Teflon-lined autoclave that had 10 ml of 1 M NaOH solution. After the autoclaves were sealed, they were heated to 120°C for 10 h in an oven. Finally, the samples were taken out, washed with deionized water and ethanol in turns, and then naturally dried in air at room temperature.

Field emission scanning electron microscope (FESEM, MAIA 3 GMU, TESCAN, Czech) was performed to observe the microstructure of the alloy, the surface morphology, and the cross-section morphology of the treated samples. Energy dispersive X-ray spectrometer (EDS, Oxford Instruments, UK) was used to analyze the elemental distribution of the treated samples. X-ray photoelectron spectroscopy (XPS, PHI-5000 Versa Probe III, ULVAC-PHI, Japan) was also conducted to obtain the chemical composition of the samples after surface treatment. X-ray diffraction (XRD, D8 ADVANCE, Bruker, Germany) was performed to characterize the phase composition of the treated and untreated samples. Here, an incident angle of 1° was adopted in the XRD experiment for the treated sample.

A saturated Ca(OH)_2_ solution was used to simulate the concrete pore solution in this study, and in order to investigate the effect of chloride ions on the corrosion resistance, the saturated Ca(OH)_2_ solution was further diluted by 3.5 wt.% NaCl solution with a volume ratio of 1 : 1. Electrochemical corrosion tests were conducted on a CHI660E electrochemical workstation, and the specimen with a surface area of 1 × 1 cm^2^ was exposed to 200 ml of simulated concrete pore solution. Here, the potential was referenced to a saturated calomel electrode (SCE) and the counter electrode was a platinum sheet. After immersion for 30 min, the electrochemical impedance spectra (EIS) were collected from 100 kHz to 100 mHz with a 5 mV sinusoidal perturbing signal at the open-circuit potential. Potentiodynamic polarization curves following the EIS test were recorded from -1.8 V to 0 V at a scanning rate of 1.0 mV·s^−1^. All the electrochemical measurements were repeated three times to ensure reproducibility. In addition, an immersion test was also carried out to further evaluate the corrosion behavior in the simulated concrete pore solution. After immersion for 24 h, the samples were taken out, rinsed with water and ethanol, and naturally dried in air. Then, their surface morphologies were observed by scanning electron microscopy (SEM).

## 3. Results and Discussion

**Figure 1:**
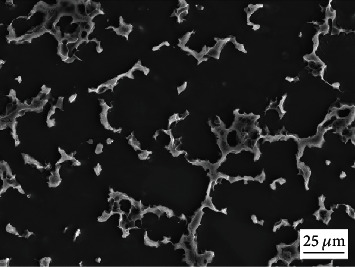
Microstructure of Mg alloys used in this study.

**Figure 2:**
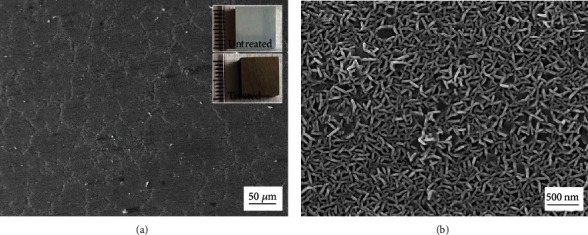
Surface morphologies of the treated Mg alloy: (a) lower magnification and (b) higher magnification. The inset shows the appearance difference between the untreated and treated sample.

**Figure 3:**
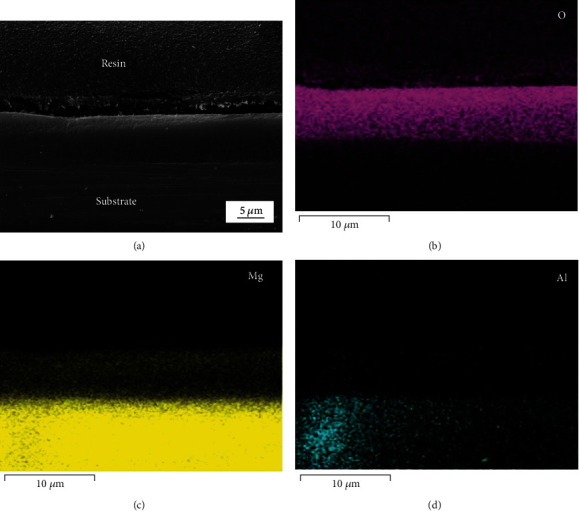
Cross-section of the hydrothermally-treated sample: (a) SEM image and (b–d) corresponding EDS elemental maps.

Mg-9.3 wt. % alloy consists of the Mg matrix and second phase, and Figure 1 shows its microstructure.  Figure 2 exhibits the macroscale and microscale surface morphologies of the treated sample. The inset in Figure 2(a) tells the appearance difference between the untreated and treated Mg alloys. It is noted that the surface of the sample turns brown after the treatment, which means that the hydrothermal process has already changed the surface of Mg alloy. As shown in Figure 2(a), the surface seems smooth when it is observed by SEM under lower magnification. Interestingly, it is further revealed under higher magnification that the hydrothermally-modified surface is covered by microsheets, which is clearly shown in Figure 2(b).  Figure 3 shows the cross-section of the treated sample. A layer of dense coating is clearly shown in Figure 3(a), whose thickness is about 5 *μ*m. Figures 3(b)–3(d) gives the EDS elemental maps of the corresponding site in the cross-section. Combined the SEM image and its EDS elemental maps, it is identified that the coating is mainly composed of element O and Mg.

**Figure 4:**
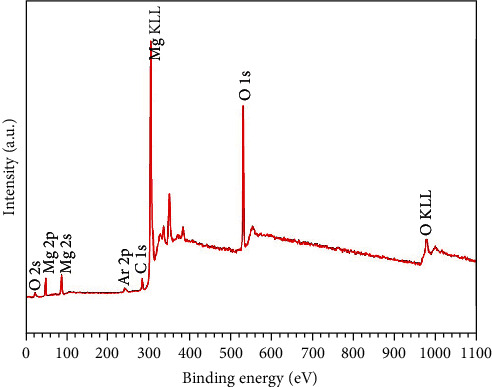
XPS survey spectra of the hydrothermal coating.

**Figure 5:**
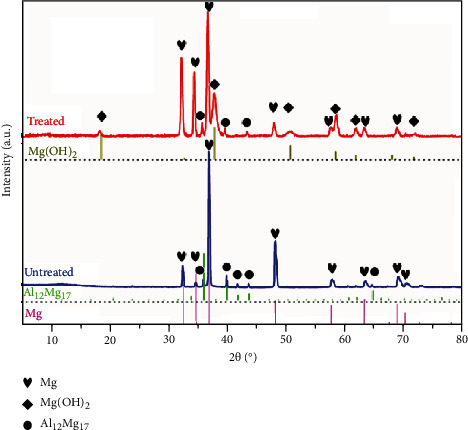
XRD patterns of the samples.

Figure 4 shows the XPS survey spectra of the top of the hydrothermal coating. In the spectra, it can be found that it is mainly composed of elements Mg and O, which is consistent with the result of EDS analysis. According to the spectra, it can be further obtained that the atomic ratio of O to Mg is about 2.58. Here, the presence of the oxygen on metals and alloys may be due to the surface oxidation and the unavoidable presence of adventitious contamination [28, 29].  Figure 5 presents the XRD pattern of the hydrothermally-treated Mg alloy, and the pattern of the untreated sample is provided here for reference. On the curve of the untreated sample, it exhibits the peaks corresponding to the Mg matrix and the second phase (Al_12_Mg_17_). Thus, it can be easily confirmed by comparison that the coating mainly consists of Mg(OH)_2_.

**Figure 6:**
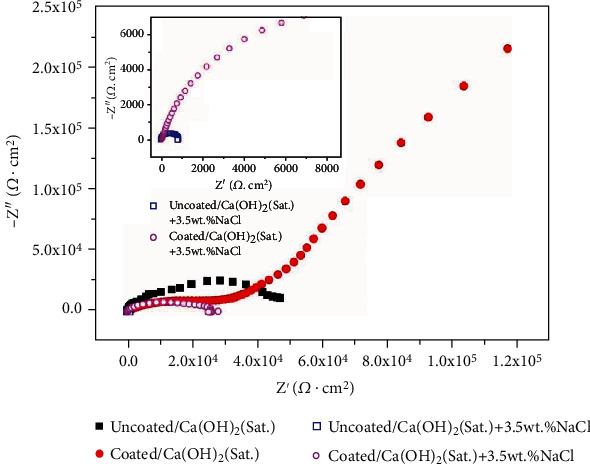
Nyquist plots of the samples in different solutions.

**Figure 7:**
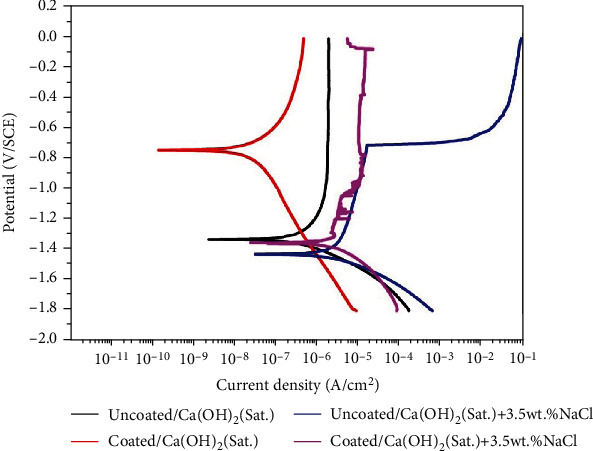
Potentiodynamic polarization curves of the samples in different solutions.

Figure 6 shows the Nyquist plots of the samples in different solutions, and the inset gives the enlarged curves of the samples immersed in Cl^−^-containing solutions. Based on the EIS theory, the polarization resistance can be derived from the following formula: *R*_*p*_ = *R*_*ω*→0_–*R*_*s*_. Here, *R*_*p*_ is the polarization resistance, *R*_*ω*→0_ denotes the zero-frequency impedance, and *R*_*s*_ represents the solution resistance [30]. In order to perform a rapid evaluation of corrosion resistance, a simplified way is adopted that the resistance at the lowest frequency in our investigation is used to substitute the polarization resistance. As shown in Figure 6, it can be found that the corrosion resistance in the saturated Ca(OH)_2_ solution is higher than that in the Ca(OH)_2_ solution diluted by 3.5 wt.% NaCl solution. After the hydrothermal treatment, the corrosion resistance is significantly improved in both kinds of test solutions.  Figure 7 presents the polarization curves of the samples in their corresponding solutions. The corrosion potential and corrosion current density are derived from cathodic Tafel region extrapolation, and their data are as shown in Table 1. For the uncoated Mg alloy, it has a higher corrosion current density in the simulated concrete pore solution with Cl^−^ than that in the simulated concrete pore solution without Cl^−^. It is known that higher corrosion current density means lower corrosion resistance. After coating, the corrosion resistance of Mg alloy has been significantly improved in either the solution with Cl^−^ or the one without Cl^−^. In the solution with Cl^−^, the coated alloy's corrosion resistance is close to that of the uncoated one in the simulated concrete pore solution without Cl^−^. In addition, compared to the uncoated sample, the coated one does not have an obvious transition potential in the investigated anodic polarization region in the solution with Cl^−^. In all, the results show that the hydrothermal coating on magnesium alloy has a protective effect as manifested by the lower corrosion current density and higher transition potential.

**Figure 8:**
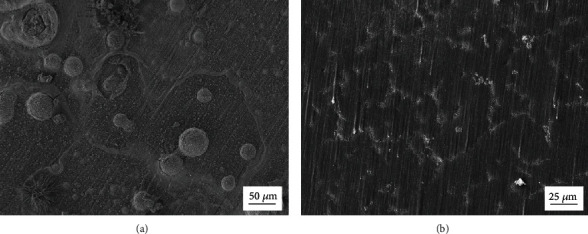
(a) SEM image of the uncoated sample after immersion for 24 h. (b) SEM image of the coated sample after immersion for 24 h.

Figure 8 shows the surface morphologies of the samples after the immersion in simulated concrete pore solutions with the addition of sodium chlorides for 24 h. As mentioned above, the solution is prepared by the mixture of saturated Ca(OH)_2_ solution and 3.5 wt. % NaCl solution with a volume ratio of 1 : 1. From the SEM images, it can be clearly seen that the surface of the uncoated sample is corroded, whereas that of the coated one still keeps intact even under the observation of higher magnification. Obviously, the result of the immersion test is in accordance with that of the electrochemical corrosion test, indicating that the hydrothermal coating can protect the Mg alloy substrate well in Cl^−^ containing simulated concrete pore solutions.

Magnesium alloys are considered for building materials in this study, and the corrosion resistance in simulated concrete pore solutions has been investigated preliminarily. To the best of our knowledge, there have been few studies on the corrosion behavior of magnesium alloys in simulated concrete pore solutions. As expected, Mg alloys used in this study have a good corrosion resistance in simulated concrete pore solutions due to the alkalinity of the solutions. But, when chloride ions are added into the simulated concrete pore solutions, the corrosion behavior of Mg alloys is significantly altered. Fortunately, the hydrothermal process can produce a layer of dense coating on Mg alloys for the retardation of the attack of chloride ions. It is known that defects will be formed more or less in the coatings at the stage of coating preparation or service, and the occurrence of galvanic corrosion will be fatal to the coating/substrate system if the coating is electroconductive. Here, the hydrothermal coating is mainly composed of Mg(OH)_2_, and compared to other metallic or conductive ceramic coatings, it can avoid the galvanic corrosion effectively. Furthermore, the hydrothermal solution used in this study is very simple, which make it a facile and economical processing for the applications of magnesium alloys in building engineering.

## 4. Conclusion

In this study, a layer of dense coating is successfully prepared on Mg-9.3 wt. % Al alloy by hydrothermal treatment. SEM discloses that the coating has a thickness of about 5 *μ*m, and the top of the coating is covered by microsheets. It is determined by the results of XRD, EDS, and XPS that the coating is mainly composed of Mg(OH)_2_. Due to the compact microstructure of the hydrothermal coating, the corrosion resistance of Mg alloy after the hydrothermal treatment is significantly improved in simulated concrete pore solution with and without the addition of chloride ions. In summary, this facile method provides a feasible way to improve the corrosion resistance of magnesium alloys for building engineering in the future.

## Figures and Tables

**Table 1:** Corrosion potential and corrosion current density determined from polarization curves.

	*E*_*corr*_ (V/SCE)	*I*_*corr*_ (A∙cm^−2^)
Uncoated/[Ca(OH)_2_ (Sat.)]	−1.343 ± 0.021	(4.06 ± 0.24) × 10^−6^
Coated/[Ca(OH)_2_ (Sat.)]	−0.839 ± 0.131	(2.71 ± 1.23) × 10^−8^
Uncoated/[Ca(OH)_2_ (Sat.)+3.5 wt.% NaCl]	−1.417 ± 0.009	(1.01 ± 0.16) × 10^−5^
Coated/[Ca(OH)_2_ (Sat.)+3.5 wt.% NaCl]	−1.371 ± 0.015	(3.95 ± 3.79) × 10^−6^

## Data Availability

Answer: Yes. Comment: The data used in this study are available from the corresponding author upon request.
